# Dr. Salvador Villalpando Hernández

**DOI:** 10.1016/j.advnut.2024.100349

**Published:** 2025-01-17

**Authors:** Steven A Abrams

**Affiliations:** Department of Pediatrics, Dell Medical School at the University of Texas at Austin, Austin, TX, United States

In this issue of *Advances in Nutrition: An International Review Journal*, we are publishing a tribute to Dr. Villalpando, who recently passed away in Mexico [1]. This tribute was lovingly written by his colleagues at the National Institute of Public Health (INSP) where he worked for over 20 y. I had the tremendous personal honor of working directly with Dr. Villalpando on a project related to iron absorption in a government-supported program to provide enhanced nutrition to small children in Mexico [2].

I was privileged to get to know him well, both professionally and personally. Truly he was a wonderful scientist and advocate for the children he cared for as well as the medical and nutritional science professionals’ community of Mexico. He graciously welcomed me and my family to INSP and into his home. We will miss him in the pediatric and nutrition community and are delighted to be able to publish this In Memoriam article describing his work and many contributions to nutrition and public health.

Although publishing “In Memoriam” articles is certainly a sad event, we welcome any suggestions for such tributes for scientists in the nutrition field who have recently passed away. We are pleased to be able to honor these deserving individuals.

## Author contributions

The sole author was responsible for all aspects of this manuscript.

## Funding

The author reported no funding received for this study.

## Conflicts of interest

The author reports no conflicts of interest.
